# Biogas Residues Improved Microbial Diversity and Disease Suppression Function under Extent Indigenous Soil Microbial Biomass

**DOI:** 10.3390/life13030774

**Published:** 2023-03-13

**Authors:** Yubin Zhao, Kai Hu, Jiadong Yu, Md. Tariful Alam Khan, Yafan Cai, Xiaoling Zhao, Zehui Zheng, Yuegao Hu, Zongjun Cui, Xiaofen Wang

**Affiliations:** 1 Institute of Agricultural Resources and Regional Planning, Chinese Academy of Agricultural Sciences, Beijing 100081, China; 2 College of Agronomy, China Agricultural University, Beijing 100193, China; 3 Institute of Environment and Sustainable Development in Agriculture, Chinese Academy of Agricultural Sciences, Beijing 100081, China; 4 School of Chemical Engineering, Zhengzhou University, Zhengzhou 450001, China

**Keywords:** soil microbial biomass, microbial community, co-occurrence pattern, fertilizer type, microbial function

## Abstract

Indigenous soil microbial biomass (ISMB) plays a key role in maintaining essential functions and biodiversity of soil health. One of the critical unknowns is how the indigenous microorganisms respond to different fertilizers which is directly related to agricultural production. Therefore, we used Mi-Seq sequencing and network analyses to compare the response of ISMB to biogas residue and chemical fertilizers. The results showed that crop production was profoundly influenced by levels of ISMB present and is further dependent on the strategy of fertilizer application. Higher ISMB primarily manifests through retention of richer microbial abundance, a balanced community structure, and tightened co-occurrence within a certain proportion of *Nitrospirae*, *Rhizophlyctidaceae*, and *Gemmatimonadetes*. Compared to chemical fertilizer, biogas residue resulted in higher production with more strongly linked nodes such as *Actinobacteria*, *Chloroflexi* and *Gemmatimonadetes*. Under the same level of ISMB, the microbial diversity was richer and co-occurrence was tighter when biogas residues were applied compared with chemical fertilizer. In addition, the higher level of ISMB with biogas residue applied had a lower abundance of potential fungal pathogens in both bulk and rhizosphere soil compared with chemical fertilizer. This study provides critical data to understand the influence of ISMB and biogas residue on soil ecological system.

## 1. Introduction

Over the past 30 years, soil microbiology has attracted a large amount of attention across many fields, not least because of the fundamental role of soil microorganisms in establishing biogeochemical cycles [1,2,3,4]. The diversity and biomass of microorganisms in soil have been used to provide rapid and accurate information concerning changes in soil quality, which is widely considered the indicator of soil fertility and ecosystem productivity [5,6,7]. In general, greater amounts of diversity and biomass lead to improved soil fertility and higher levels of crop production. Soil microbial diversity and biomass both play key roles in maintaining the essential functions of soil health, and the interaction between the two factors directly affects the growth of crops [8,9,10]. Thus, there should be a close relationship between the biomass and diversity of soil microorganisms. The effects of microbial diversity and biomass have been described and reviewed in relation to many physical and chemical properties, such as pH, temperature, salinity, and porosity [11,12,13]. However, the mechanism of interaction of microbial characteristics as indigenous soil microbial biomass (ISMB) and its effect on microbial diversity is usually neglected in most studies on microbial diversity.

In terms of natural processes, the fertility of farmland soil slowly degrades and the reduction of the biomass of microorganisms may require tens or hundreds of years. In recent years, the soil has been increasingly affected by human activity, and the destruction caused by this human factor has shortened time of degradation of number of microorganisms [14]. Examples include the application of high temperature sterilization technology in vegetable greenhouses or a substantial amount of pesticides [15]. Such management measures affect the soil and result in the loss of microbial diversity or biomass not only of pathogenic bacteria but also of beneficial bacteria, which may influence the biogeochemical processes occurring in soil [16,17]. In the case of these measures, different fertilizers are applied for agricultural production. Chemical fertilizers and biogas residue fertilizers have different properties, leading to questions on whether the indigenous microorganisms in the soil respond to different type of fertilizers and to what extent the diversity of microorganisms may be changed, which is a important issue that is directly related to agricultural production. 

Chemical fertilizers provide available nutrients for microbial growth. However, studies found that the excessive use of chemical fertilizers in recent years has deleteriously changed the microbial community [18]. When the biomass of soil microorganisms differs, the question becomes how to adjust the community structure in the face of a relative lack or presence of sufficient nutrients to reach an appropriate balance. Thus, new types of fertilizer needed to be applied to balance the deranged and homogeneous soil microbial community. A substantial amount of previous research indicates that different types of organic fertilizer provide a variety of inorganic and organic nutrients for crops while fertilizing the soil and improving the soil microbial ecosystem [19,20,21,22]. Many beneficial microorganisms, such as *Bacillus* and *Pseudomonas*, are contained in different organic fertilizers and may help the native microorganisms to rebuild a healthy environment and improve nitrogen use efficiency and crop production [23,24]. Biogas residues are prospective organic fertilizer sources and substitutes for chemical fertilizers in agricultural cultivation [25]. These fertilizers contain macroelements (N, P, K, Ca and Mg) and microelements (Fe, Al, Cu and Zn), which can improve and stimulate plant growth [26,27]. Biogas residue is also a potential way to rebuild microbial biomass and improve soil functions [28,29,30]. Thus, the response of soil microbial diversity to different ISMB is important to enhance our understanding of biological assemblage in agricultural soil, particularly following the application of chemical and biogas residue fertilizer.

The objective of this study was to comprehensively evaluate relationships among soil microbial diversity, ISMB, and fertilizer type. A statistical analysis was performed on the level of ISMB, fertilizer type, and soil habitat to explore their contributions to the soil physicochemical properties, microbial diversity, and structure. Then, a network analysis was used to explore the symbiotic relationship of bacteria and fungi under the level of ISMB, different fertilizers, and soil habitats. Finally, the plant pathogens were predicted using the fungal functional guild. The results of this study provide novel insights on how biogas residue and ISMB balance ecological and production benefits. 

## 2. Materials and Methods

### 2.1. Experimental Design 

**Figure 1:**
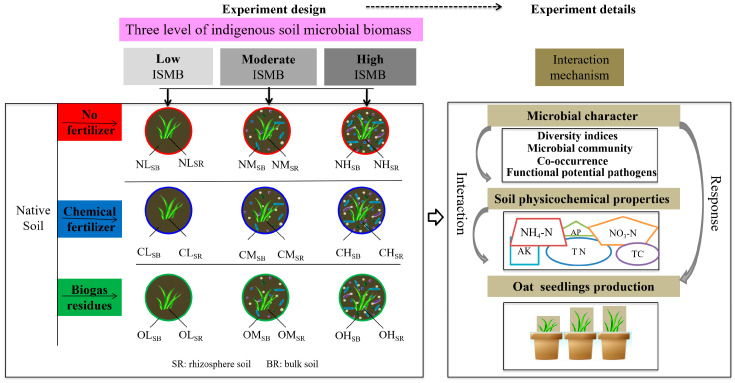
Experimental design.

In this study, different levels of ISMB were created using different autoclaved treatment: non-sterile soil (high level [H]), mixture of half non-sterile soil and half completely sterile soil (moderate level [M]), and completely sterile soil (low level [L]). Three fertilizer types (no fertilizer [N], chemical fertilizer [C], biogas residues [O]) and two soil habitats (bulk soil [SB] and rhizosphere soil [SR]) were studied (Figure 1). All 19 experimental treatments (3 levels of ISMB × 3 type of fertilizer × 2 type of soil habitat and one native soil) were conducted on three replicates for each soil sample (100 g soil_dry matter_). For the O treatment, 1000 g dry soil and 0.2 g biogas residues were used, and 0.1 g/kg N was added using biogas residues. For the C treatment, chemical fertilizer (nitrogen, phosphorous, and potassium) was applied using the same amount of nitrogen, phosphorous, and potassium as that of the O treatment (0.1 g/kg N, 12.53 mg/kg P, 20.81 mg/kg K). The physical and chemical properties, total bacterial and fungal diversity, and composition of the soil were then analyzed after one cropping (30 days).

### 2.2. Soil and Plant Sampling 

Soil was collected in 2017 from the Shangzhuang Experimental Station in Beijing, China, after planting corn from 2015 to 2017. Then, the soil was subjected to the 19 treatments, and each treatment contained 3 pots (15 × 10 × 10 cm, height × top diameter × bottom diameter), representing a total of 57 pots. The soil properties of the samples were as follows: the total nitrogen content was 0.45 ± 0.04 g kg^−1^, the organic carbon content was 31.60 ± 1.33 g kg^−1^, the NH_4_^+^-N content was 0.10 mg kg^−1^, the NO_3_^−^-N content was 2.65 mg kg^−1^, the available potassium content was 197.178 ± 8.68 mg kg^−1^, and the available phosphorus content was 27.41 ± 0.11 mg kg^−1^. In December 2017, before experiments were conducted in a light incubator (28 ± 2 °C) at the Agricultural University of China, several fresh soil samples were sterilized at 121 °C for 15 min using an autoclave (MLS-3020, Sanyo, Japan). O treatment in this study used one type of biogas residue. The biogas residues were derived from oat straw and cow manure. Biogas residues properties were as follows: the total nitrogen content was 2.43 ± 0.04 g kg^−1^, the total phosphorus content was 3.07 ± 0.25 g kg^−1^, and the total potassium content was 5.1 ± 0.01 g kg^−1^. The biogas residue used in this study was type I biological fertilizer produced by Beijing Liuhe Biotechnology Company, which comprised functional microbial strains with an effective viable count ≥200 million/g and a lignite adsorption matrix. The total nitrogen content was 8.05 g kg^−1^, the total phosphorus content was 26.57 g kg^−1^, and the total potassium content was 2.19 g kg^−1^. The chemical fertilizer used in this study was composed of urea (CON_2_H_4_), Ca(H_2_PO_4_)_2_·H_2_O and K_2_SO_4_.

### 2.3. Treatment of Oat Seed

In order to prevent the seed bacteria from disturbing the soil microorganisms, the oat seed was treated with 75% ethanol for 30 s and 2% sodium hypochlorite for 10 min prior to conducting the experiments. The seeds were then washed five times with sterilized distilled water. 

### 2.4. Soil Physicochemical Properties and Crop Yield

Mineral nitrogen pools (NO_3_^−^ and NH_4_^+^) present in the soil were extracted by adding a 50-mL aliquot of 1 M KCl to 10 g of fresh soil. The resulting suspension was shaken at 80 rpm for 1 h at 25 °C. Quantification was performed using colorimetry with at least two blanks in each series (Model 410 Flame Photometer, Sherwood, Cambridge, UK). The total nitrogen (TN) and organic carbon (TC) content of the soil was measured by elemental analysis (Elementar Vario MAX CHN/CNS, Hanau, Germany). Because these soils were free from carbonates, the total carbon content of the soil was equivalent to the soil organic carbon content. The available phosphorus (AP) was quantified using the NaHCO_3_^−^ extraction method and a spectrophotometer (7200, Shanghai, China), and the available potassium (AK) was quantified using the NH_4_OAc extraction method and a flame photometer (FP640, Shanghai, China). The oat seedling was oven-dried at 80 °C for 24 h. The AE_N_ (Agronomic efficiency of applied N) (kg/kg), the agronomic efficiency of the applied N, was calculated using the crop yield difference between the two fertilizer treatments and the no-fertilizer treatment conditions, respectively [31].

### 2.5. DNA Extraction

Soil microbial DNA was extracted from a 500 mg sample from each of the 57 soil samples, and the total DNA concentration in each sample was determined with an E.Z.N.A.^®^ Soil DNA Kit (Omega Bio-Tek, Norcross, GA, USA) according to the manufacturer’s instructions. The final DNA concentration and purity were determined using a NanoDrop 2000 UV-vis spectrophotometer (Thermo Scientific, Wilmington, NC, USA), and DNA quality was examined via 1% agarose gel electrophoresis.

### 2.6. Amplicon Generation and Illumina MiSeq Sequencing 

Bacterial consortia were further analyzed by sequencing the V3–V4 hypervariable region of the 16S rRNA gene. The V3–V4 region was amplified using universal primers 338F and 806R (338F: 5′-ACTCCTACGGGAGGCAGCA-3′ and 806R: 5′-GGACTACHVGGGTWTCTAAT-3′). PCR reactions were performed in triplicate using the following reaction mixture (total volume: 20 μL): 4 μL of 5× FastPfu Buffer, 2 μL of dNTPs (2.5 mM), 0.8 μL of each primer (5 μM), 0.4 μL of FastPfu Polymerase and 10 ng of template DNA. The hypervariable regions of the fungal 18S rRNA gene were amplified with primers 817F and 1196R (817F: 5′-TTAGCATGGAATAATRRAATAGGA-3′ and 1196R: 5′-TCTGGACCTGGTGAGTTTCC-3′) using a thermocycler PCR system (GeneAmp 9700, ABI, NY, USA). The PCR reactions were conducted using the following program: 3 min of denaturation at 95 °C was followed by 35 cycles of 30 s at 95 °C, 30 s at 55 °C (annealing step) and 45 s at 72 °C (elongation step), with a final extension step of 10 min at 72 °C. The PCR products were purified using a 2% agarose gel. The products were extracted from the gel using an AxyPrep^TM^ DNA Gel Extraction Kit (Axygen Biosciences, Union City, NJ, USA) and quantified using a QuantiFluor™-ST fluorometer (Promega, Madison, WI, USA) according to the manufacturers’ instructions. The sequencing data have been deposited in the National Genomics Data Center database under accession number PRNJA004295.

### 2.7. Sequencing Data Processing

Raw FASTQ files of bacteria and fungi were demultiplexed and quality-filtered using QIIME (version 1.17). First, the reads were truncated at any site receiving an average quality score <20 over a 50 bp sliding window. Second, exact barcode matching was performed, and primer matching was conducted in which a two-nucleotide mismatch and reads containing ambiguous characters were removed. Finally, sequences with overlaps longer than 10 bp were merged according to their overlap sequence. 

Operational units (OTUs) were clustered with a 97% similarity cutoff using UPARSE (version 7.1 http://drive5.com/uparse/, 14 November 2019), and chimeric sequences were identified and removed using UCHIME. The taxonomy of each 16S rRNA gene sequence was analyzed by the Ribosomal Database Project (RDP) Classifier (http://rdp.cme.msu.edu/) against the silva128/16s_bacteria 16S rRNA database and Unite 7.0/its_fungi as references with a confidence threshold of 70%.

### 2.8. Statistical Analyses

Data were analyzed using a free online platform, Majorbio I-Sanger Cloud Platform (www.i-sanger.com). A rarefaction curve obtained using the sequencing data was flat, indicating the sequencing data were sufficient. Rarefaction curves of the observed richness (S_obs_) were calculated in MOTHUR (version v.1.30.1) using 1000-fold resampling without replacement. Estimates of diversity were based on evenly rarefied OTUs and included the Chao indices, which are observed community richness indices and the Shannon indices, which are community evenness indices. PICRUSt and FUNGuild were used to predict the metagenomes using the 16S-based and 18S-based OTU tables via the free online platform available on Majorbio Cloud Platform (www.majorbio.com), and Bray–Curtis distances (for Beta diversity comparison between OTU or PICRUSt KO abundances across samples) were calculated [32]. 

Analysis of similarities (ANOSIM) and Multivariate analysis of microbial diversity was performed by community composition analysis (R version 3.3.3), principal coordinate analysis (PCoA), Student’s t-test (equal variance) and Fisher’s exact test (R version 3.3.3). Permanova and paired sample t-test for different soil habitats were analyzed by SPSS 20.

The network analysis [33] was performed at the genus (abundant > 0.1) and family (abundant > 0.001) level of the bacteria and fungi. To explore all the pairwise associations, correlation scores (Spearman correlation, Pearson correlation, Kullback–Leibler dissimilarity, Bray–Curtis dissimilarity and mutual information) were calculated by SPSS. The *p* values of the five methods were integrated by the Brown method, and only significant correlations (correlation coefficient. >0.6; *p* < 0.05) were retained for the downstream procedure. The resulting correlations were imported into the Gephi platform and then visualized by the Frucherman–Reingold algorithms. The topology property parameters in the network were calculated by Gephi. The cut off value of the hub of anode was set at 0.2 to explore the putative key hubs of each network [34,35]. Soil physicochemical property data were analyzed by Microsoft Excel 2010 pro and SPSS 20.

## 3. Results

### 3.1. Oat Seedling Production and Soil Physicochemical Properties 

**Figure 2:**
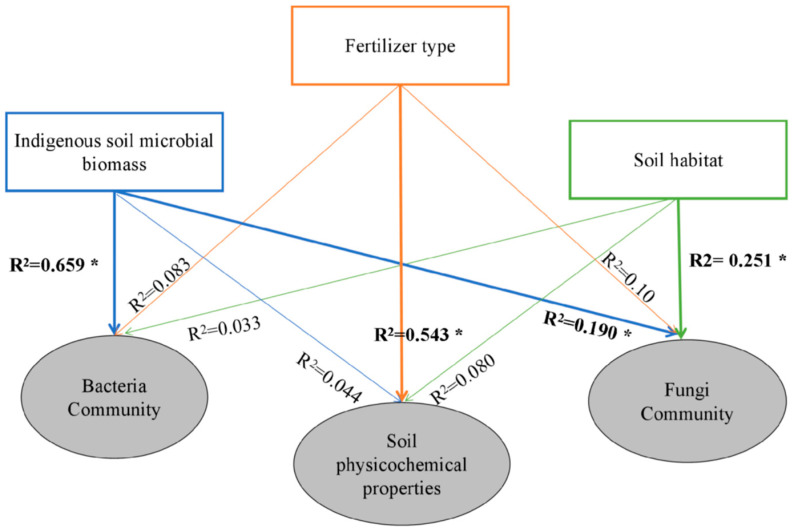
Effect of soil microbial biomass, fertilizer type, and soil habitats treatment on the differentiations of soil physicochemical properties and bacterial and fungal communities based on PERMANOVA. Value in bold indicates a significant difference at *p* < 0.05. * *p* < 0.05.

The ISMB and fertilizer type treatments induced significant (*p* < 0.05) changes in the production of oat seedlings as ascertained by dry matter and stem length (Table 1). Higher production correlated with the higher level of ISMB in no fertilizer and chemical and biogas residues treatments, and the highest amount of production took place at a high level of ISMB with biogas residues. The AE_N_ (Agronomic efficiency of applied N) of biogas residues treatment was higher than the chemical fertilizer treatment in all three levels of ISMB soils. The ratio of AE_N_ (O/C) increased 1.60–6.27 folds from low ISMB to high ISMB (Figure S1). The soil physicochemical properties differed significantly between fertilizer type (*p* = 0.001, R^2^ = 0.543) but not between ISMB (*p* = 0.888, R^2^ = 0.044) and soil habitat (*p* = 0.245, R^2^ = 0.080), based on PERMANOVA (Figure 2) [36]. The ISMB treatments exhibited noticeable changes (*p* < 0.05) in all soil physicochemical properties with the exception of available potassium. The soil habitat only induced significant (*p* < 0.05) changes in the available potassium, ammonia nitrogen, and nitrate nitrogen (Table 1). The changes in soil physicochemical properties along with ISMB level are shown in Figure S3. The application of high and moderate levels of ISMB increased soil’s total nitrogen (average 0.50% and 0.49%, respectively) and total organic carbon (average 30.68 and 37.22 g/kg, respectively), compared with the application of low levels of ISMB (average 0.45; 28.73 g/kg) treatment and surpassed that of native soil (0.45%; 31.60 ± 1.15 g/kg). Moderate and high levels of ISMB (217.56 and 218.41 mg/kg, respectively) decreased the amount of available potassium compared with that of low ISMB (221.02 mg/kg) treatment, which surpassed the concentrations found in native soil (197.18 ± 10.63 mg/kg). In addition, the available potassium of SR samples was lower than that of SB samples in the three levels of ISMB. The changes in available phosphorus were complex: in the H treatment, samples with the biogas residues had higher available phosphorus (107.58 mg kg^−1^) and NO_3_^−^-N (0.59 mg kg^−1^) for the OH_SR_, which far exceeded the levels in native soil (27.41 ± 0.14 mg/kg) (Figure S2). We also compared the difference of soil physicochemical properties between biogas residues and chemical fertilizer in three level ISMB soils. In general, the increase index of TC and AP was 5.42–39.55% and 8.50–301.67%, and the decrease index of TN and AK was 3.11–19.064% and 8.91–25.30%. Therefore, the higher level of ISMB with biogas residues was important for maintaining the soil physicochemical properties, particularly for the accumulation of organic carbon and available phosphorus, and the efficient use of nitrogen and available potassium.

### 3.2. Alpha Diversity and βeta Diversity of Bacteria and Fungi

#### 3.2.1. Alpha Diversity

ISMB induced significant (*p* < 0.05) changes in the alpha diversity of both bacteria and fungi based on Shannon and Chao indices, and the fertilizer type and soil habitat did not induce significant (*p* > 0.05) changes in alpha diversity (Table 2). Treatment of the bacteria and fungi with low levels of ISMB significantly correlated with moderate and high ISMB treatments for the two indices described above ([Shannon] *p* < 0.05, and [Chao] *p* < 0.05), but the Shannon index of fungi in soil treated with low and moderate levels of ISMB did not change significantly (*p* > 0.05) (Table S1). No fertilizer and chemical and biogas residues were significantly correlated with each for either bacteria or fungi (Table S1). The two alpha diversity indices of bulk soil and rhizosphere soil have a significant correlation for bacteria (Shannon = 0.929, *p* < 0.05 and Chao = 0.847, *p* < 0.05) but not for fungi (Table S2).

The richness (Chao) and evenness (Shannon) alpha diversity indices of bacterial and fungal diversity are shown in Table S3. The Chao and Shannon indices of bacterial diversity decreased with low ISMB treatment compared with those of native soil. The Chao and Shannon indices of bacterial diversity with moderate and high ISMB treatments increased and could reach a level similar to that of native soil. In addition, OH_SR_ produced the highest Chao index (3091.09), which was 10.69% higher than that of native soil (2792.47). Interestingly, at the same level of ISMB, the Chao and Shannon indices following treatment with biogas residues were almost the highest compared with chemical fertilizer, and the increase index of Chao was from 2.96–98%. Using the same fertilizer, the SR samples could almost increase Chao and Shannon indices compared with those of SB samples. Notably, a highly complex phenomenon was observed for low ISMB samples: in the rhizosphere soil, NL_SR_ increased the Chao and Shannon indices, CL_SR_ decreased the Chao and Shannon indices, and OL_SR_ increased the Chao index and decreased Shannon index. 

For the three ISMB treatments, almost all the samples treated with different types of fertilizer exhibited a decrease in the Chao and Shannon fungal diversity indices in SR samples compared with SB samples, and the indices increased in SR samples following H treatments. The highest Chao index of OH_SR_ was 459.65, which was 19.88% higher than that of native soil (383.44). Moreover, the fungal diversity indices with biogas residues almost decreased compared with the chemical fertilizer samples. This result showed that microbial diversity of bacteria was easier to restore comparing with fungi, and the biogas residue conducive to the restoration of soil microbial diversity is based on a certain amount of soil indigenous microorganisms.

#### 3.2.2. Beta Diversity

**Figure 3:**
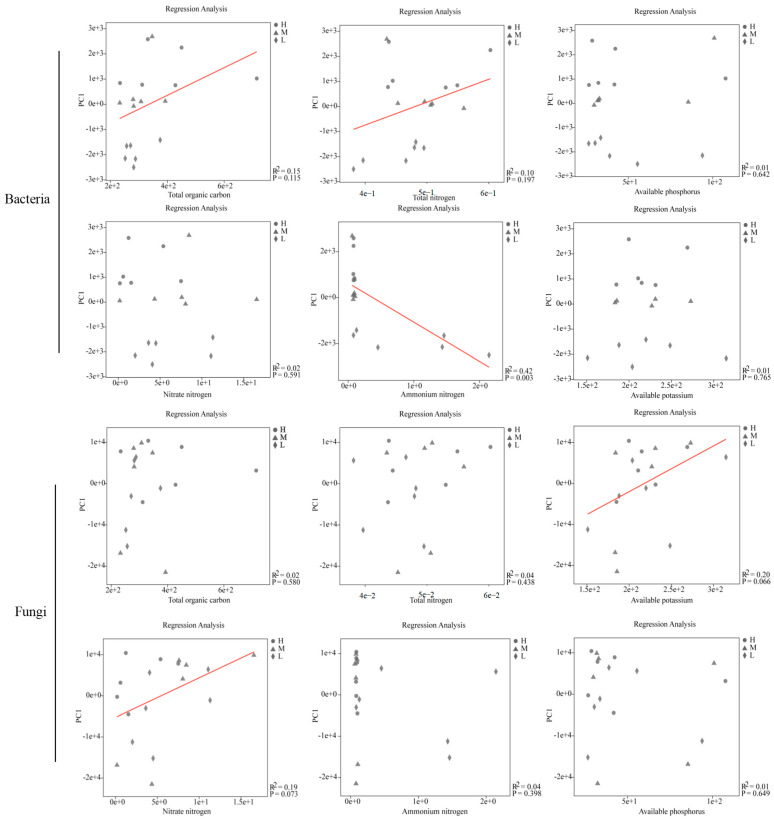
Linear regression analysis of the physicochemical properties of soil and microbial OTUs at low, moderate, and high soil microbial biomass levels [L], [M] and [H]).

An analysis of similarities (ANOSIM) showed that the level of ISMB produced the most substantial effects on diversity of bacterial and fungal communities with regard to the OTU level compared with application of different types of fertilizer and soil habitat effects. PCoA (principal co-ordinates analysis) of the unweighted UniFrac distance between samples revealed a stronger degree of clustering of established microbial communities of both bacteria and fungi according to their level of ISMB (Figure S2). The bacterial community differed significantly between ISMB (*p* = 0.001, R^2^ = 0.659), and the fungal community differed significantly between ISMB (*p* = 0.008, R^2^ = 0.251) and soil habitat (*p* = 0.004, R^2^ = 0.190; Figure 2). Linear regression analyses of the physicochemical properties and microbial OTUs are shown in Figure 3. In general, the soil physicochemical properties (total organic carbon, total nitrogen, available phosphorus, and nitrate nitrogen) positively correlated with relative abundance of bacteria and fungi. However, the ammonia nitrogen was negatively associated with relative abundance of bacteria (*p* < 0.05). According to the significant results of statistical analyses, microbial community, co-occurrence, and functional potential pathogens group changes affected in order of ISMB levels, fertilizer type and soil habitat are presented in detail as follows:

### 3.3. Soil Bacterial and Fungal Community 

**Figure 4:**
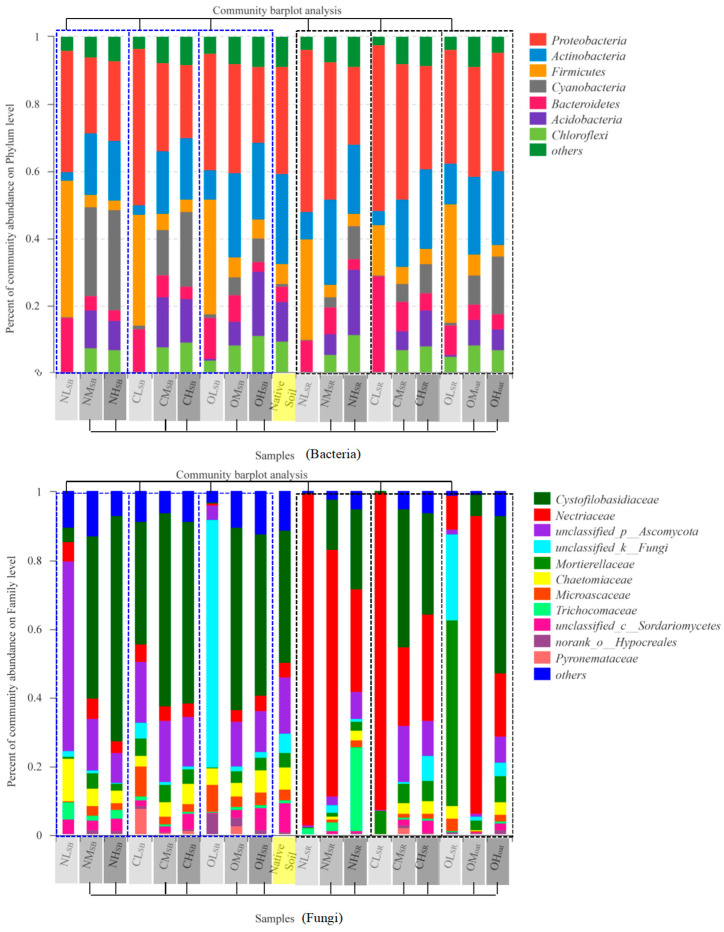
Community composition of native soil and restored microcosms. Microcosms established in bacterial communities (at the phylum level) and fungal communities (at the family level) of soils treated with different fertilizers.

Differences in the microbial community were analyzed by community bar plots (Figure 4). Bacteria had 3624 rarefied OTUs with a sequence number of 877246 and an average length of 487.16. Fungi had only 711 rarefied OTUs with a sequence number of 1561195 and an average length of 255.68.

#### 3.3.1. ISMB Level Changes the Soil Bacterial and Fungal Community

Without considering the effects of plants, the observed bacterial diversity of low ISMB treatment was the lowest within the three ISMB levels, and only *Proteobacteria*, *Firmicutes* and *Bacteroidetes* were the abundant bacteria. The abundance of the three bacteria was much higher than that of native soil, from 6.04% (native soil) to 14.98–40.55% (NL_SB_, CL_SB_ and OL_SB_), from 4.58% (native soil) to 8.83–15.56% (NL_SB_, CL_SB_ and OL_SB_), and from 31.69% (native soil) to 34.73–46.58% (NL_SB_, CL_SB_ and OL_SB_), respectively. The primary genera present in the low ISMB treatments included *Bacillus* (Firmicutes), *Massilia* (*Proteobacteria*) and *Tumebacillus* (*Firmicutes*). The moderate and high ISMB treatments showed there were seven major bacteria: in addition to the *Firmicutes*, *Proteobacteria,* and *Bacteroidetes*, the abundance of *Actinobacteria*, *Chloroflexi*, *Cyanobacteria,* and *Acidobacteria* also became significant bacteria (Figure 4). The abundance of seven predominant bacteria in moderate and high ISMB treatments were similar to the native soil and became more evenly distributed. Moderate and high ISMB treatments decreased *Actinobacteria* and *Proteobacteria* and increased *Cyanobacteria* compared to native soil. The principal genera present in the moderate ISMB treatments included *Massilia* (*Proteobacteria*) and *Pseudarthrobacter* (*Actinobacteria*). The main genera present in the high ISMB treatments soil included *Microcoleus* (*Cyanobacteria*) and *Pseudarthrobacter* (*Actinobacteria*). 

The observed fungi diversity of low ISMB treatments showed drastic changes. The abundance of *Ascomycota* was 93.17% in NL_SB_, which was greatly increased with native soil (51.12%). CL_SB_ did not significantly change the abundance in comparison to native soil. OL_SB_ compared with native soil; *Basidiomycota* decreased rapidly, *Ascomycota* decreased to 26.23%, and *unclassified_k_Fungi* increased to 71.98%. Moderate and high ISMB treatments did not significantly affect the soil fungi community, which was keeping composition stable and similar to the native soil, where *Cystofilobasidiaceae*, *Nectriaceae*, *unclassified_p_*Ascomycota, *unclassified_k_Fungi*, *Chaetomiaceae*, and *Microascaccae* were the dominant community (Figure 4).

#### 3.3.2. Fertilizer Type Changes the Soil Bacterial and Fungal Community

Fertilizer type was the secondary factor that did not present significant changes in the soil bacterial (R^2^ = 0.083; *p* = 0.684) and fungal community (R^2^ = 0.109; *p* = 0.482) after the indigenous microbial level (Figure 2). The microbial community was more similar to the native soil with biogas residues applied than chemical fertilizer, especially in moderate and high ISMB. Altogether, the predominant abundance of *Cyanobacteria* and *Proteobacteria* in the bacterial community of the soil treated with biogas residues, *Acidobacteria* and *Actinobacteria* in the bacterial community of the soil treated with chemical fertilizer, and *Chloroflexi* and *Actinobacteria* in the bacterial community of the soil treated with no fertilizer was significantly different from the predominantly abundant bacteria present in native soil. With regard to the effects of fertilizers on the fungal community, significant differences included the predominant abundance of *Basidiomycota* and *Zygomycota* in soil treated with biogas residues and *Ascomycetes* in soils treated with chemical fertilizer.

#### 3.3.3. Soil Habitat Changes the Soil Bacterial and Fungal Community

Soil habitat was another secondary factor that affected the soil bacterial and fungal community after the indigenous microbial level. Plants had greater influence on soil fungi than bacteria because of the rhizosphere effect. The effects of SR samples on bacteria were similar with the SB samples, and the distributions of seven dominant bacteria were more even and stable.

However, the changes for the fungi of SR samples were complex following the low, moderate, and high ISMB level. For low ISMB level, *Ascomycota*, with an abundance of 99.59%, became the predominant microorganisms in NL_SR_. Notably, the abundance of the pathogenic fungus *Fusarium* (a common pathogen causing wilt) and *unclassified_f__Nectriaceae* increased for SR samples (NL_SR_, CL_SR_, OL_SR_) comparing with SB samples (NL_SB_, CL_SB_, OL_SB_). NL_SR_ produced abundance values of 87.41% and 1.86% for *Fusarium* and *unclassified_f__Nectriaceae*, respectively. CL_SR_ had lower *Basidiomycota*, *Fusarium* and *unclassified_f__Nectriaceae* comprised 16.02% and 76.26% of the fungal community, respectively. The OL_SR_ treatment changed the soil fungal community significantly, *Zygomycota* reached an abundance of 54.29%, becoming the most dominant species in the soil, with *Ascomycota* comprising only 19.91%. *Fusarium* and *unclassified_f__Nectriaceae* were effectively controlled at 5.03% and 4.34%. However, all the abundance values of *Fusarium* and *unclassified_f__Nectriaceae* of NL_SR_, CL_SR_ and OL_SR_ were higher than those of native soil, 1.59% and 0.14%. Thus, the interaction of plants and microorganisms was important for assembling the new microbial community.

SR samples in moderate and high ISMB levels produced different results when different fertilizers were applied. The abundance of *Fusarium* and *unclassified_f__Nectriaceae* increased significantly to 70.49% (8.27% and 62.22%, respectively) for NM_SR_ and 84.93% (15.07% and 84.93%, respectively) for OM_SR_ and was only 18.05% (9.28% and 8.77%, respectively) for CM_SR_. In addition, *Nectriaceae* significantly increased, showing an abundance of 71.74% and 86.79% for NM_SR_ and OM_SR_, respectively, while the abundance of *Nectriaceae* for CM_SR_ was only 22.84%. The pathogenic fungi (*Fusarium* and *unclassified_f__Nectriaceae*) were well controlled and only had an abundance of 16.88% (3.84% and 13.04% for *Fusarium* and *unclassified_f__Nectriaceae*, respectively) for OH_SR_. However, their abundance increased to 29.52% (16.28% and 13.24% for *Fusarium* and *unclassified_f__Nectriaceae*, respectively) for CH_SR_ and 28.83% (2.36% and 26.37% for *Fusarium* and *unclassified_f__Nectriaceae*, respectively) for NH_SR_. Therefore, biogas residue was a relatively safe treatment for soil with high ISMB of microbial communities. 

### 3.4. Co-Occurrence Pattern for Soil Bacteria and Fungi Community 

**Figure 5:**
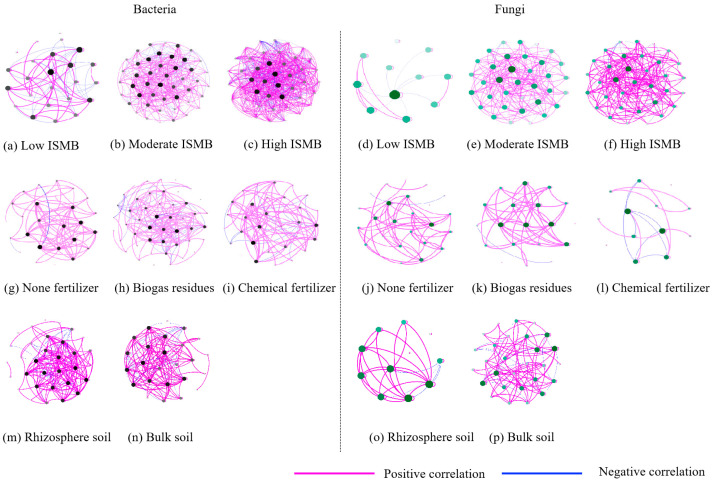
These networks visualize different soil microbial biomass treatment effects on the co-occurrence pattern between bacterial taxa at genus level with relative abundances greater than 0.1%, and fungal taxa at family level with relative abundances greater than 0.001% in soils. The node size is proportional to abundance of taxa, and the edges are colored according to interaction types. Positive correlations are labeled with pink, and negative correlations are labeled with blue.

To determine the effects of ISMB on microbial associations, six networks were constructed for the soil bacterial and fungal communities affected by low, moderate, and high levels of ISMB, respectively (Figure 5a–f). The co-occurrence for both soil bacterial and fungal communities became more complex with higher levels of ISMB. Compared with that of the H treatment, the clustering coefficients of network of the M and the L treatments decreased by 0.269 and 0.194 for the bacteria, respectively (Table 3a), indicating that the soil bacterial associations were loose under the lower level of ISMB. Positive correlation coefficients increased with the increase in levels of ISMB. The positive correlation coefficients differed only slightly and were 68.37%, 67.65%, and 64.63% for the H, M, and L treatments, respectively. The results showed that with higher levels of microorganisms, the amount of mutually beneficial cooperation increased. These results were also supported by the data on number of nodes and edges. The number of nodes of bacteria in networks of the M and the H treatments increased by 100% and 91.30%, respectively, compared with those of L treatment. Key hub analysis further suggested that the bacterial taxa differed in three levels of ISMB (Table S5). A bacterial taxon within the phyla *Chloroflexi*, *Gemmatimonadetes*, and *Nitrospirae* (*Nitrospira*) was most connected in H networks, while it was not linked to any other microbial taxa in the networks of L and M treatments. The phylum *Acidobacteria* in M treatment, *Bacteroidetes* and *Firmicutes* in L treatment were the microbial taxa found to be strongly linked nodes, but they were not linked to any other microbial taxa. This indicated that the co-occurrence pattern of a low level of ISMB depended on the presence of close evolutionary relationships among species. 

For the fungal community, the changes of clustering coefficient were the same as those of bacteria (Table 3b). The clustering coefficient of M and L treatments decreased by 0.435 and 0.542, respectively, compared with that of H treatment. In contrast, the positive correlation coefficients decreased with higher levels of ISMB unlike those of bacteria. The positive correlation coefficients were 61.87%, 89.42%, and 66.67% for the H, M, and L treatments, respectively. The number of nodes of fungi in the networks of the M and H treatments increased 2.18-fold and 2.73-fold, respectively, compared with those of the L treatment. The fungal taxa within family *Rhizophlyctidaceae* were most connected in H networks, while it was not linked to any other microbial taxa in the networks of L and M treatments (Table S6). 

Similarly, specific networks that focused on different fertilizer types were created (Figure 5g–l; Table 3a,b). Clustering coefficients of the network of bacteria and fungi in O treatment both increased compared with those of N treatment, while the two both decreased in C treatment compared with those of N treatment. The results indicate that the associations of soil microorganisms were tightened more following the application of biogas residues but were more loose following the application of chemical fertilizer compared with no fertilizer treatment. The application of biogas residues and chemical fertilizers decreased positive correlation coefficients of bacteria and fungi, and the application of biogas residues significantly decreased positive correlation coefficients of bacteria, and the application of chemical fertilizer significantly decreased the positive correlation coefficients of fungi. The focused network analyses revealed that the number of nodes of bacteria in networks of the O and C treatments increased by 67.86% and 17.86%, respectively, compared with the networks of N treatment. In contrast, the number of nodes of fungi in networks of the O and C treatments decreased by 24% and 48%, respectively, compared with the networks of N treatment. Key hub analysis further suggested that the bacterial and fungal taxa varied among different fertilizer types (Tables S7 and S8). The phyla *Actinobacteria* and *Chloroflexi* in O treatment were the microbial taxa found as strongly linked nodes, but they were not linked to any other microbial taxa in N and C treatment. A bacteria taxon within the phyla *Chloroflexi* and *Gemmatimonadetes* was most connected in O networks, while it was not linked to any other microbial taxa in the networks of N and C treatments. The phylum *Verrucomicrobia* was a unique microbe in C treatment owing to its strongly linked nodes (Table S7). The families *Lasiosphaeriaceae* and *Trichocomaceae* were most connected in O networks and not linked to any other microbial taxa in the networks of N and C treatments (Table S8). 

Clustering coefficients of the network of bacteria in SB treatment decreased but increased compared with the SR treatment of fungi (Figure 5m–p; Table 3a,b). The number of nodes of bacteria and fungi in the networks of the SB decreased by 21.88% and increased by 1.64-fold, respectively, compared with the networks of SR treatment. The results indicated that associations of soil microorganisms were tightened more among bacteria but were looser among fungi in bulk soil compared with those in rhizosphere soil. Interestingly, SB decreased the positive correlation coefficients of both bacteria and fungi compared with those of SR, which indicated that the soil microorganisms would be more mutually beneficial because of the guidance of oat roots. Key hub analysis further suggested that the bacterial and fungal taxa differed in two soil habitats. The phyla *Chloroflexi* and *Actinobacteria* in SR and SB treatments were the specific microbial taxa found as strongly linked nodes, respectively (Table S9). The phyla *unclassified_k__Fungi* and *Zygomycota* (*Mortierellaceae*) were the unique microbes in SB treatment found as strongly linked nodes (Table S10). 

### 3.5. Variations of Fungal Functional Potential Pathogen Groups 

The application of chemical fertilizers resulted in a higher relative abundance of potential animal pathogens and plant pathogens when the level of ISMB increased in both SB samples and SB samples. However, the application of biogas residues and no fertilizer resulted in a lower relative abundance of potential animal pathogens and plant pathogens along with the increase in levels of ISMB. When the levels of ISMB were moderate and high, the OM_SR_ and OH_SR_ decreased 30.56% and 59.42% relative abundance of potential pathogens compared with those of the CM_SR_ and CH_SR_ samples, which could occur because of the higher content of total organic carbon, nitrate nitrogen, and available potassium (Figure S5). This indicated that biogas residues had higher disease resistance than chemical fertilizer as long as soil is not extremely deficient in microorganisms. The Spearman correlation heatmaps of fungi showed that the total organic carbon, nitrate nitrogen, and available potassium negatively correlated with the pathogenic *Nectriaceae (Fusarium* and *unclassified_f__Nectriaceae)* (Figure S6). With no fertilizer, the level of ISMB became the sole single factor to control potential pathogens. The relative abundance of potential animal pathogens and plant pathogens was highest in NL_SR_ sample. The predictive functional profiling of the bacterial communities under low, moderate, and high ISMB level is also shown in Figure S4. Low ISMB level have a different KO pathway comparing with the moderate and high ISMB level. “KO2014: iron complex outermembrane recepter protein”, “KO3406: methyl-accepting chemotaxis protein”, and “KO2529: LacI family transcriptional regulator” were the three less abundant pathways in low ISMB treatment. Moderate and high ISMB had higher abundance in K08884 (serine/threonine protein kinase, bacterial [EC:2.7.11.1]), KO2033, KO2033, KO2034, and KO2035 (peptide/nickel transport system protein) (Table S4). The ISMB level changed the metabolic pathway of bacteria which may affect the abundance of potential pathogen groups. Given the limitations of PICRUSt and FUNguild functional prediction, there is still great potential for exploration due to some unexplored functions. However, combing the plant yield, soil physicochemical property, and microbial diversity, we could still probably speculate on the potential effect of different fertilizers on the function of soil microorganism.

## 4. Discussion

### 4.1. Microbial Community Formation and Co-Occurrence Pattern under Different Levels of Soil ISMB

The results indicated that ISMB plays a key role in stability and restoration of soil ecology. A high level of ISMB resulted in a more responsive microbial community (in terms of their relative abundance), which was associated with the highest levels of crop production, nutrient availability, and lowest relative abundance of potential fungal pathogens following both chemical fertilizer and biogas residue compared with that of low ISMB. It is well known that soil health is determined by a number of complex biochemical processes, which are driven by different microorganisms [37]. Much research has shown that microbial diversity results in better soil environments: the interactions between microorganisms, such as Actinomyces species and *Trichoderma harzianum*, favorably interfaced with two established antagonists (*Bacillus cereus* and *B. subtilis*) to inhibit the growth of some plant pathogenic fungi [38,39]. The interaction of these beneficial microorganism results in soil that is resilient to risk of disease, with sufficient stability to resist adverse factors, such as a reduction in the abundance of *Fusarium* [40]. It was clearly apparent that one cluster of microorganisms can easily become the absolute dominant fungi when the level of ISMB is low, such as the abundance of *Ascomycota* of 93.17% in NL_SB_, and *unclassified_k_Fungi* of 71.98% in OL_SB_, resulting in an imbalance in the soil microbial community. Therefore, the balance of mutualistic symbiosis or mutual inhibition among microorganisms would be destroyed. Previous research also reached similar conclusions, which indicated that following disturbances, an ecosystem with a higher microbial biomass may have a higher capacity to sustain the ecological processes through microbiological buffering [7,41]. Therefore, higher levels of ISMB indicate the presence of a richer microbial community, and the increased number of interactions could be a prerequisite for formation of a healthy soil ecosystem.

Moreover, we found that the diversity of microorganisms was not sole indicator of soil health. In our study, the moderate ISMB treatment, although it had a similar microbial community to that of the high ISMB treatment, displayed a pattern of tightened co-occurrence that was inoperative within lower amounts of *Nitrospira* and Rhizophlyctidaceae. Although biogas residues can provide beneficial microorganisms and nutrients, these factors were unable to aid in the restoration of soil ecology at lower levels of ISMB. This may be because the low level of ISMB within relative richness of nutrients, which does not create intense competition of microorganisms, resulted in a reduced interaction between microorganisms. The microorganisms that reproduce faster or are more adapted to environmental impacts, such as *Firmicutes* in this study, will quickly become the dominant microorganisms and occupy the soil. Moreover, the unknown microorganisms maybe stimulated in sterilized environmental samples to rebuild the microbial community [42]. However, when rich levels of ISMB were already present in the soils, the capacity of soil to resist risk increased with a high buffer capacity. There were not enough nutrients for the microorganisms, and thus, the competition or other types of interactions would have proceeded. Functional beneficial microorganisms in biogas residues that compete with indigenous microorganisms for nutrients forces them to actively reproduce, consolidate themselves, accelerate the regrowth of microorganisms, and maintain a new balance [43]. In our study, the more tightened co-occurrence of both bacteria and fungi with biogas residues compared with that of chemical fertilizer also proved this phenomenon. Thus, high levels of ISMB could maintain the balance of soil ecosystem not only by presence of rich microorganisms but also by tightened co-occurrence.

Fertilizer type in agriculture is an indispensable factor that needs to be fully considered. Higher levels of ISMB could reduce the release of immobilized nutrients to enhance soil nutrient conservation [7]. Rich diversity and a high level of soil ISMB aided in the maintenance of soil fertility [44]. One factor that cannot be ignored is that the application of different types of fertilizers could change soil nutrient cycle. In our study, the application of chemical and biogas residues significantly changed physicochemical properties of soil. The application of biogas residues in soil with a high level of ISMB results in higher available phosphorus, organic carbon, and NO_3_^−^-N content because of the rich abundance of microorganisms, such as *cyanobacteria* and *Trichocomaceae*, that are involved in related biochemical reactions. Previous research provides solid evidence that N fixation and certain groups of diazotrophic taxa will be largely suppressed as increasing amounts of chemical fertilizers have become the mainstay in agricultural production [45]. Thus, microbial function could decline without the stress of survival because of excess application of chemical fertilizer. Therefore, methods of the application of biogas residues can play a better role than chemical fertilizer to rebuild the soil ecosystem.

### 4.2. Interactions of Plant and Soil Microorganisms under Different Levels of Soil ISMB 

**Figure 6:**
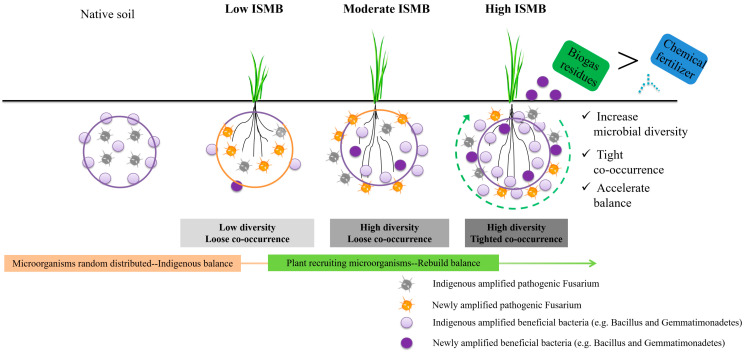
Microbial balance rebuilt under different ISMB following application of different types of fertilizer.

This study showed that in the presence of higher levels of ISMB, the oat seedlings produce more, and the abundance of soil microorganisms is richer. A close relationship between plant and microorganisms was demonstrated by Oates et al. [46], who showed that the composition of plant community can strongly influence soil microbial community. When vegetation was restored to the soil bacterial community structure, the co-occurrence pattern was changed in a karst rocky desertification area [47]. Similarly, the root exudates of maize and soybean also changed the soil bacterial community structure [48]. Once plant growth is threatened, plants will seek help from soil microorganisms and gather beneficial microorganisms for disease resistance [8]. These studies all indicated that plant root exudates may act as activators for microorganisms, which can stimulate different physiological and biochemical reactions of microorganisms. Thus, the indigenous microbial balance of native soil would be fragmented when the plant was considered. The interaction of plant microorganisms is the focus of research under different ISMB levels following application of different types of fertilizer (Figure 6). When the level of ISMB is low, as shown in this study, one cluster of species such as *Nectriaceae* that responds positively will dominate the microbial community. This condition could destroy the balance of soil microorganisms, resulting in low diversity, loose co-occurrence, and low crop production. Some research has indicated that higher amounts of *Nectriaceae* are dangerous for soil health and can cause a severe disease of black root rot [49]. Plant secretions have different effects on microorganisms, and microorganisms respond to them in varying ways with different rates of reactions and propagation. However, when the ISMB level is higher, even if the microorganisms that are slow to respond to plants do not gather and reproduce as fast as those microorganisms that respond quickly, there are enough members of indigenous populations to balance the newly added microorganisms, which will not lead to an imbalance of the soil micro-ecological environment. Moreover, we found that although moderate levels of ISMB increase the microbial diversity, they lose the co-occurrence. Only when the ISMB reach their highest level are diversity and co-occurrence both maintained in an excellent state. Of course, it is easier to maintain a balance if biogas residue is applied with beneficial microorganisms compared with chemical fertilizers. The genus *Fusarium* includes many pathogenic species causing a wide range of plant diseases [50,51] so that we discuss the interactions between *Fusarium* and other key bacteria to analyze the character of different fertilizers. Our study clearly found that the key bacteria that maintain co-occurrence patterns of biogas residues have a negative relationship with the *Fusarium* (Table 4). Also supporting this argument is the knowledge that *Bacillaceae* are abundant in soils created during organic farming and could be used as the first line of defense against soil-borne fungal pathogens, particularly some *Fusarium* [52,53]. Previous studies indicated that chemical fertilizer clearly disconnects the dependency of plants from many plant-enhancing microbial processes to deleteriously change the degree of microbial diversity [33]. In our study, the disconnect caused by chemical fertilizer may be achieved through a decrease in the degree of microbial diversity, which further changes the microbial co-occurrence pattern. In addition, most key bacteria maintain the co-occurrence pattern of a positive relationship of chemical fertilizers with the *Fusarium* (Table 4). Therefore, high ISMB combined with biogas residues was important to create a beneficial environment for plant growth. Different types of plant secretions vary in their effects on microbial recruitment. This study just researched the interaction between one soil and one type of crop under different amounts of microbial biomass. We suggest more opportunities to study the interaction with different microbial biomass in varying soil types and crops in more detail, so that specific fertilizers could be produced with the plant recruitment microorganisms for different crops, which can be applied to uphold the balance of soil ecosystem and continuously produce crops. 

## 5. Conclusions

This study demonstrates that the diversity and co-occurrence patterns of soil microorganisms are profoundly influenced by levels of ISMB present and are further dependent on the strategy of application of fertilizer and soil habitat. When the levels of ISMB were low, the recovery of the microorganisms, particularly the fungi, was poor, and the co-co-occurrence pattern was loose. Importantly, although moderate and high levels of ISMB could recover the diversity of bacteria and fungi to the native level, only the high level of ISMB tightened soil microbiome associations. The application of biogas residues increased soil microbial abundance, decreased abundance of pathogenic microorganisms, and enhanced the co-occurrence patterns, which was more conducive to soil recovery compared with soil that had been treated with chemical fertilizer. In addition, the associations of soil microbiome were tightened more by bacteria but were looser among fungi in bulk soil compared with rhizosphere soil. In conclusion, we propose that when sufficient levels of indigenous microorganisms are present in the population of soil microorganisms, they favorably affect agricultural soil ecosystem function and sustainability. Thus, the protection of soil indigenous microorganisms should be considered superior to restoration of soil. Moreover, for soil in which the balance of microorganisms has been destroyed, biogas residues was one preferred choice to regenerate soil ecosystem. Given the critical roles of soil microorganisms in soil functionality, the results from this study provide novel insight to understand how biogas residue and ISMB balance ecological and production benefits. 

## Figures and Tables

**Table 1:** Effect of soil microbial biomass, fertilizer type, and soil habitats treatment on changes of physicochemical properties and oat seedling production based on general linear model.

		ISMB	Fertilizer Type	Soil Habitat	Fertilizer Type × ISMB	Fertilizer Type × Soil Habitat	ISMB × Soil Habitat	Fertilizer Type × ISMB × Soil Habitat
Total nitrogen	F	19.02	58.05	0.77	6.43	10.32	13.91	2.12
	P	0.00	0.00	0.39	0.00	0.00	0.00	0.10
Ammoniumnitrogen	F	7891.65	2494.42	22.01	2494.78	16.34	621.95	680.60
	P	0.00	0.00	0.00	0.00	0.00	0.00	0.00
Nitrate nitrogen	F	6111.05	11,753.84	22,388.83	2753.62	115.86	2420.89	435.78
	P	0.00	0.00	0.00	0.00	0.00	0.00	0.00
Organic carbon	F	380.85	126.21	0.00	118.45	257.84	181.66	178.39
	P	0.00	0.00	0.98	0.00	0.00	0.00	0.00
Available phosphorus	F	6740.72	197,601.14	3.11	137,813.05	66,588.33	74,504.35	33,461.68
	P	0.00	0.00	0.10	0.00	0.00	0.00	0.00
Available Potassium	F	1.08	553.12	236.64	17.83	9.19	23.97	10.59
	P	0.36	0.00	0.00	0.00	0.00	0.00	0.00
Dry matter	F	12.97	9.26		0.20			
	P	0.00	0.00		0.94			
Stem length	F	12.30	5.79		0.42			
	P	0.00	0.01		0.80			

*p* < 0.05 indicates a significant difference. The value of F represents the significance of the entire fitting equation. The larger the value of F, the higher the reliability of the model.

**Table 2:** General linear model of soil microbial biomass, fertilizer type, and soil habitat on the microbial alpha diversity indices (Chao and Shannon).

	Alpha Diversity	Treatment	F	P
Bacteria	Chao	Indigenous soil microbial biomass	95.48	0.00
		Fertilizer type	0.16	0.85
		Soil habitat	0.00	0.98
	Shannon	Indigenous soil microbial biomas	38.67	0.00
		Fertilizer type	0.36	0.70
		Soil habitat	0.09	0.77
Fungi	Chao	Indigenous soil microbial biomas	16.54	0.00
		Fertilizer type	0.05	0.95
		Soil habitat	1.79	0.20
	Shannon	Indigenous soil microbial biomas	3.79	0.05
		Fertilizer type	0.17	0.85
		Soil habitat	3.59	0.08

*p* < 0.05 indicates a significant difference. The value of F represents the significance of the entire fitting equation. The larger the value of F, the higher the reliability of the model.

**Table 3:** (**a**) Topological indices of each network of bacteria in Figure 5. (**b**) Topological indices of each network of fungi in Figure 5.

(**a**)								
	**ISMB Level**			**Fertilizer Type**			**Soil Habitat**	
	**Low**	**Moderate**	**High**	**N**	**O**	**C**	**SR**	**SB**
Clustering coefficient	0.5	0.43	0.7	0.71	0.72	0.69	0.75	0.73
Network density	0.32	0.36	0.6	0.29	0.19	0.24	0.37	0.51
Number of nodes	23	46	44	28	47	33	32	25
Number of edges	82	371	566	108	207	124	185	152
Average path length	2.09	1.75	1.448	2.83	2.79	2.51	1.97	1.81
Positive correlation coefficient (%)	64.63	67.65	68.37	98.15	77.78	88.71	84.32	78.29
Negative correlation coefficient (%)	35.37	32.35	31.63	1.85	22.22	11.29	15.68	21.71
(**b**)								
	**ISMB Level**			**Fertilizer Type**			**Soil Habitat**	
	**Low**	**Moderate**	**High**	**N**	**O**	**C**	**SR**	**SB**
Clustering coefficient	0.21	0.32	0.76	0.73	0.76	0.62	0.65	0.66
Network density	0.44	0.35	0.65	0.24	0.36	0.37	0.67	0.28
Number of nodes	11	35	41	25	19	13	11	29
Number of edges	24	208	535	73	62	29	37	114
Average path length	1.82	1.76	1.4	2.82	1.53	1.65	1.28	2.36
Positive correlation coefficient (%)	66.67	89.42	61.87	98.63	98.39	75.86	89.19	84.21
Negative correlation coefficient (%)	33.33	10.58	38.13	1.37	1.61	24.14	10.81	15.79

**Table 4:** Pearson’s correlation analysis between the key bacteria in different type (no fertilizer, chemical fertilizer, and biogas residues) and *Fusarium* and *Unclassified_f__Nectriaceae*.

		*Fusarium*		*Unclassified_f__Nectriaceae*	
		Correlation coefficient	P	Correlation coefficient	P
No fertilizer	*Adhaeribacter*	0.72	0.11	−0.36	0.49
	*Bacillus*	0.54	0.27	−0.01	0.98
	*Bdellovibrio*	0.68	0.14	−0.27	0.61
	*Devosia*	0.63	0.18	−0.13	0.80
	*Ensifer*	0.19	0.71	−0.17	0.74
	*Flavisolibacter*	0.42	0.41	−0.19	0.72
	*Massilia*	0.79	0.06	−0.26	0.63
	*norank_f__Chitinophagaceae*	0.45	0.37	−0.31	0.55
	*Paenibacillus*	0.58	0.23	0.39	0.45
	*Paucimonas*	0.66	0.15	−0.28	0.60
	*Rhizobium*	0.90	0.02	−0.16	0.76
Chemical fertilizer	*Adhaeribacter*	0.06	0.92	0.23	0.66
	*Bacillus*	−0.13	0.81	0.10	0.85
	*Brevundimonas*	0.56	0.25	0.17	0.75
	*Chthoniobacter*	0.14	0.80	0.52	0.29
	*Devosia*	0.55	0.26	0.85	0.03
	*Ensifer*	−0.16	0.76	0.78	0.07
	*Massilia*	0.21	0.69	0.90	0.01
	*Paenibacillus*	0.37	0.47	0.62	0.19
	*Paenisporosarcina*	0.59	0.22	0.19	0.72
	*Pedobacter*	0.37	0.47	0.52	0.29
	*Rhizobium*	0.16	0.76	0.86	0.03
	*unclassified_f__Oxalobacteraceae*	−0.08	0.89	0.66	0.15
Biogas residues	*Bacillus*	−0.37	0.47	−0.54	0.27
	*Devosia*	−0.11	0.84	0.17	0.75
	*Ensifer*	−0.08	0.88	0.32	0.54
	*Massilia*	−0.10	0.85	−0.58	0.22
	*norank_c__Gemmatimonadetes*	−0.31	0.55	−0.66	0.15
	*norank_f__Cytophagaceae*	−0.41	0.42	−0.55	0.26
	*norank_o__Acidimicrobiales*	−0.02	0.98	−0.59	0.22
	*norank_o__AKYG1722*	−0.12	0.82	−0.45	0.37
	*Paenibacillus*	−0.45	0.38	−0.64	0.17
	*Paenisporosarcina*	−0.45	0.38	−0.56	0.25
	*Rhizobium*	−0.01	0.99	0.15	0.77

## Data Availability

All data will be made available upon reasonable request.

## Supplementary Materials

The following supporting information can be downloaded at: https://www.mdpi.com/article/10.3390/life13030774/s1, Figure S1: Oat production of different treatments. Figure S2: Abundance, βeta diversity, and distance calculated at the OTU level for three groups of altered bacterial and fungi communities. Figure S3: Physicochemical properties of soil for different treatments. Figure S4: Heatmap of KO pathways of bacteria of the functional prediction by PICRUSt 2 under low, moderate and high ISMB level (L, M, H) (the most abundant 20 KO pathways). Figure S5: Variations of fungal functional groups of potential animal pathogen and plant pathogen in different treatments. Figure S6: Spearman correlation heatmaps of bacteria (phylum) and fungi (family). Heatmap: TC (Total organic carbon), TN (total nitrogen), AK (available potassium) and AP (available phosphorus), NN (Nitrate nitrogen), AN (Ammonia nitrogen) (the most abundant 20 species). Table S1: ADONIS variation test of microbial alpha diversity treated with different level of ISMB based on the Bray-Curtis distance matrix. Table S2: Paired Samples Test of SB and SR on alpha diversity index of bacteria and fungi. Table S3: Treatment effects on microbial alpha diversity. Table S4: information of treatment for the Supplementary Figure S3. Table S5: Topological indices of network under low, moderate and high ISMB level for bacteria in Figure 5. Table S6: Topological indices of network under low, moderate and high ISMB level for fungi in Figure 5. Table S7: Topological indices of network under none, chemical and biogas residues three fertilizer type for bacteria in Figure 5. Table S8: Topological indices of network under none, chemical and biogas residues three fertilizer type for fungi in Figure 5. Table S9: Topological indices of network of bulk soil and rhizosphere soil for bacteria in Figure 5. Table S10: Topological indices of network of bulk soil and rhizosphere soil for fungi in Figure 5.
