# Supplementary material for: Biogas Residues Improved Microbial Diversity and Disease Suppression Function under Extent Indigenous Soil Microbial Biomass
Source: Life (Basel). 2023 Mar 13;13(3):774. doi: 10.3390/life13030774 (PMC10055779; doi:10.3390/life13030774)
Supplement: Supplementary file 1 [file life-13-00774-s001.zip › life-2187585-supplementary.pdf]

Manuscript Title: Biogas residues improved microbial diversity and disease suppression function under extent indigenous soil microbial biomass

Yubin Zhao [1,2], Kai Hu [2], Jiadong Yu [3], Md. Tariful Alam Khan [2], Yafan Cai [4], Xiaoling Zhao [4], Zehui Zheng [2], Yuegao Hu [2], Zongjun Cui [2] and Xiaofen Wang [2,*]

1 Institute of Agricultural Resources and Regional Planning, Chinese Academy of Agricultural Sciences, Beijing 100081, China

2 College of Agronomy, China Agricultural University, Beijing 100193, China

3 Institute of Environment and Sustainable Development in Agriculture, Chinese Academy of Agricultural Sciences, Beijing 100081, China

4 School of Chemical Engineering, Zhengzhou University, Zhengzhou 450001, China

* Correspondence: wxiaofen@cau.edu.cn

Figure S1 Oat production of different treatments. [L], [M] and [H] means three levels of low, moderate, and high soil microbial biomass. [N], [C], and [O] means no fertilizer, chemical fertilizer and organic fertilizer. SB means bulk soil. SR means rhizosphere soil.

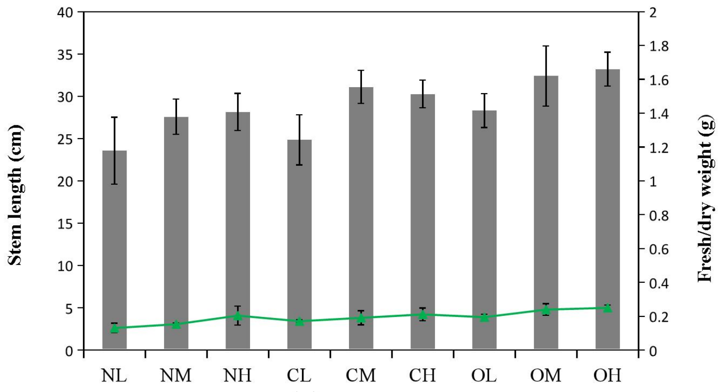

Treatments	Agronomic efficiency of applied N AE_N (kg/kg)	Ratio of AE_N O/C
CL	4.03	/
CM	3.77	/
CH	0.73	/
OL	6.43	1.60
OM	8.63	2.29
OH	4.60	6.27

(a)

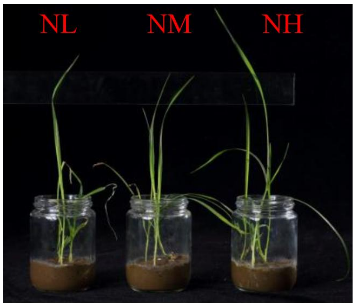

(b)

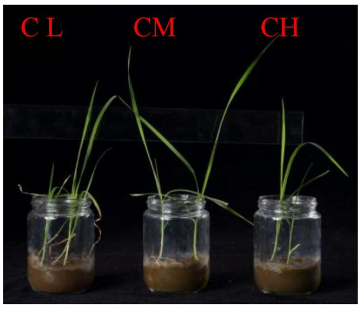

(c)

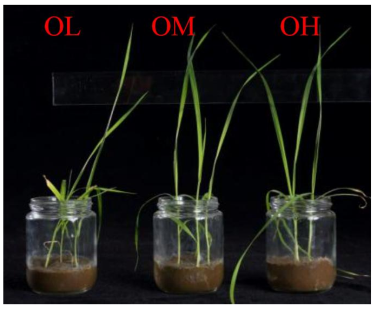

(d)

Figure S2 Abundance, βeta diversity, and distance calculated at the OTU level for three groups of altered bacterial and fungi communities. PCoA of the Bray－Curtis similarity measures representative differences in community structure of control microcosms at low, moderate, and high soil microbial biomass levels [L], [M] and [H]) for three fertilizers (no fertilizer [N], chemical fertilizer [C], organic fertilizer [O]). SB means bulk soil. SR means rhizosphere soil.

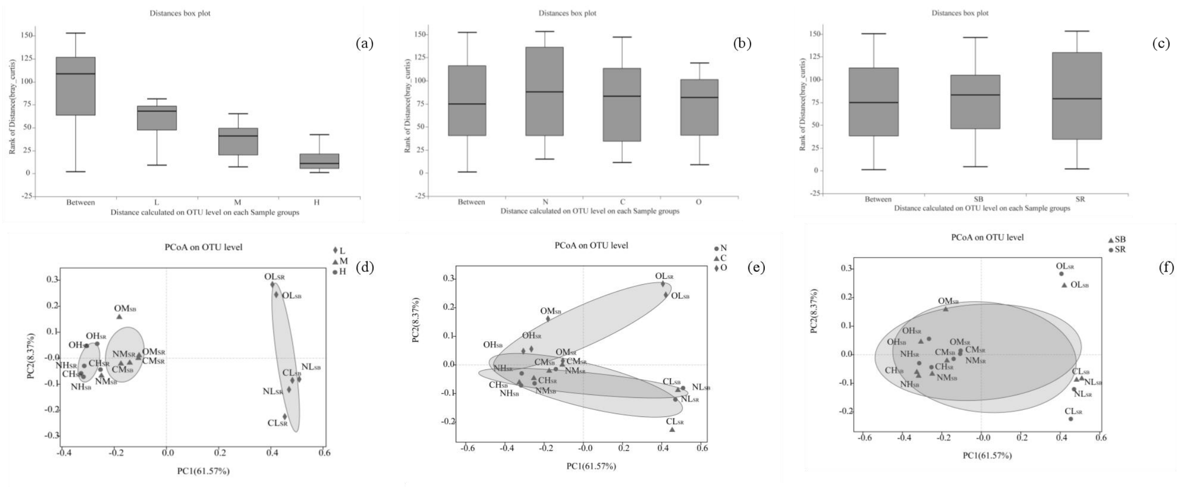

Bacteria

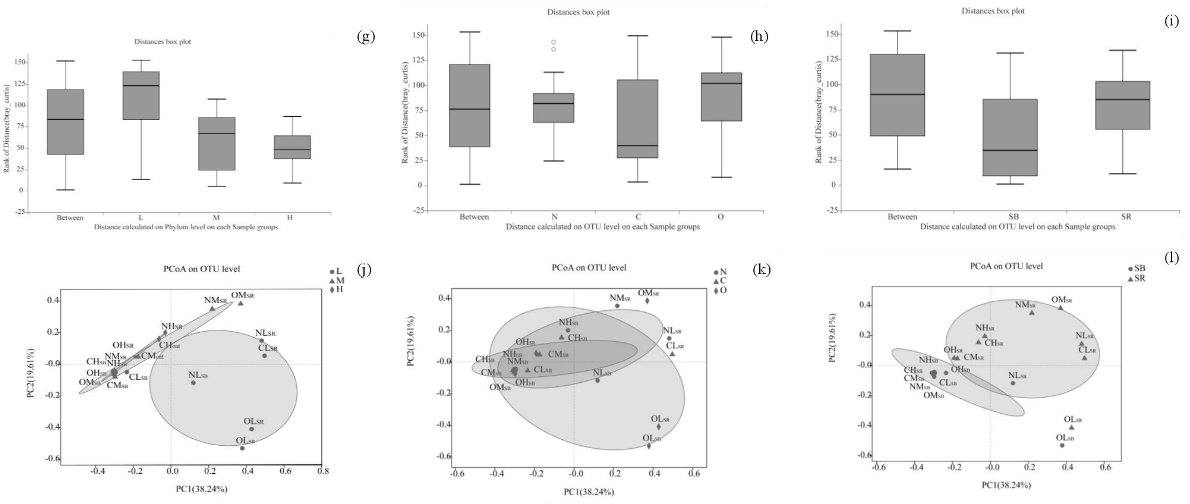

Fungi

Figure S3 Physicochemical properties of soil for different treatments. [L], [M] and [H] means three levels of low, moderate, and high soil microbial biomass. [N], [C], and [O] means no fertilizer, chemical fertilizer and organic fertilizer. SB means bulk soil. SR means rhizosphere soil.

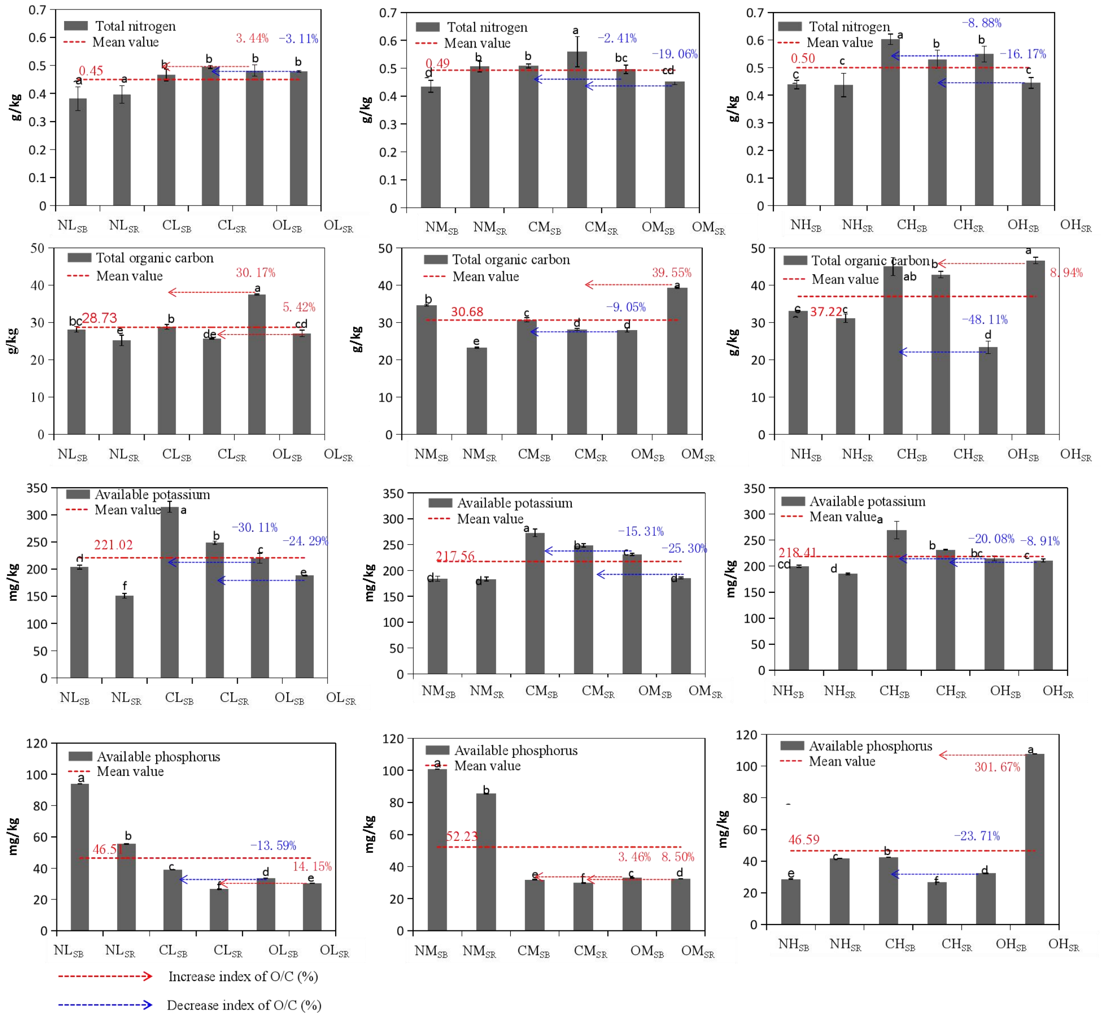

Figure S4 Heatmap of KO pathways of bacteria of the functional prediction by PICRUSt 2 under low, moderate and high ISMB level (L, M, H) (the most abundant 20 KO pathways)

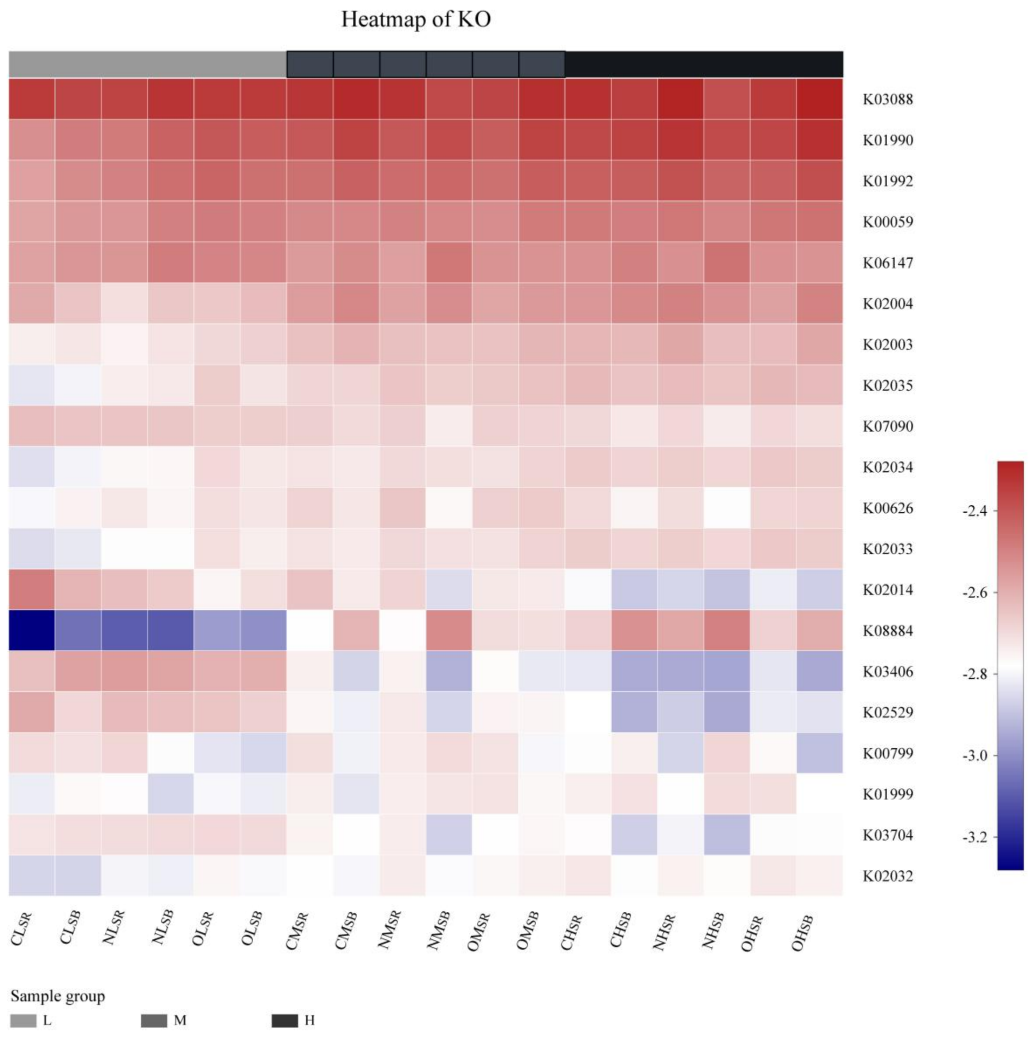

Figure S5 Variations of fungal functional groups of potential animal pathogen and plant pathogen in different treatments

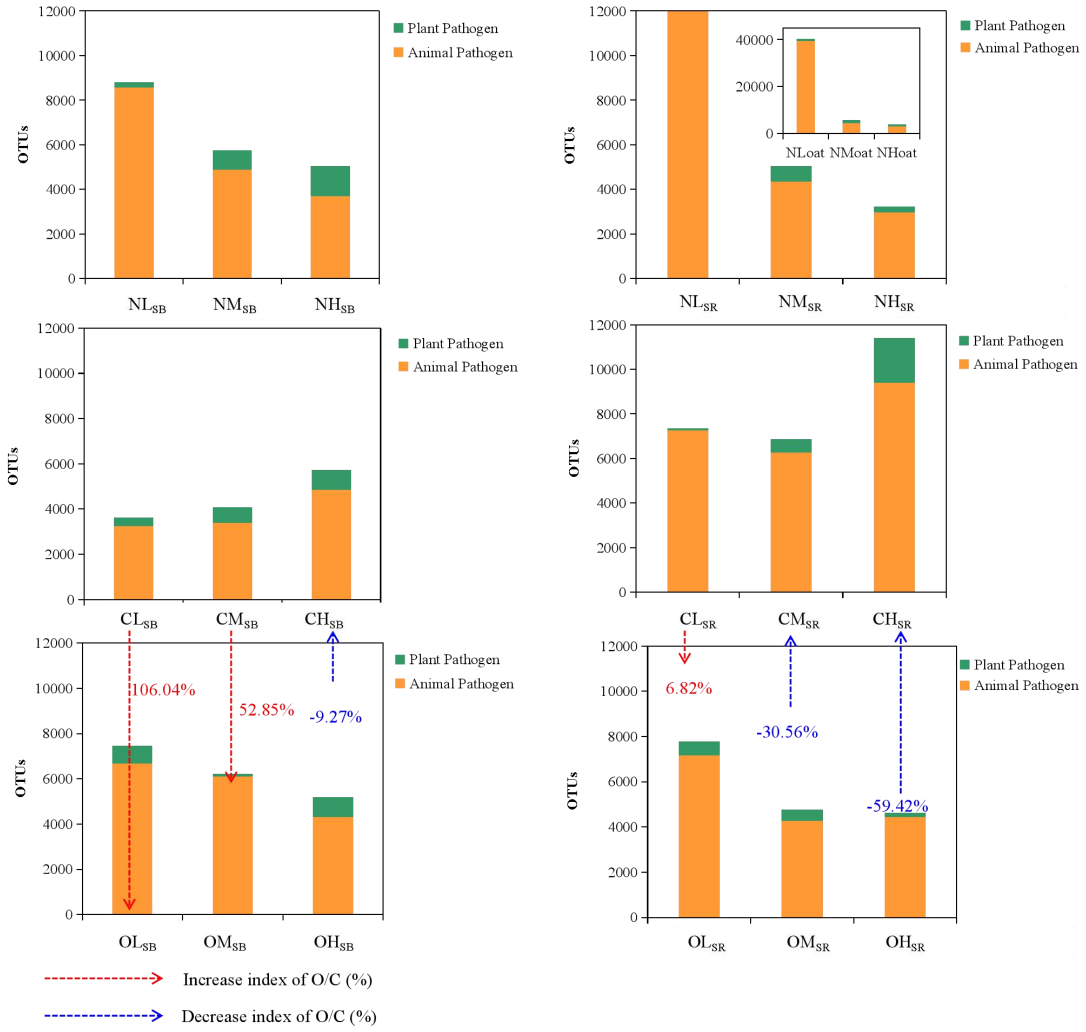

Figure S6 Spearman correlation heatmaps of bacteria (phylum) and fungi (family). Heatmap: TC (Total organic carbon), TN (total nitrogen), AK (available potassium) and AP (available phosphorus), NN (Nitrate nitrogen), AN (Ammonia nitrogen) (the most abundant 20 species).

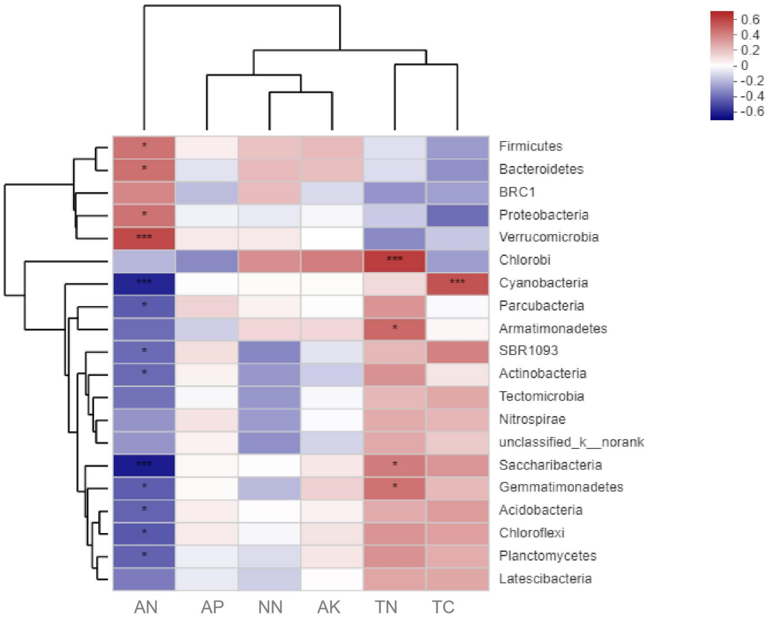

(a) Bacteria

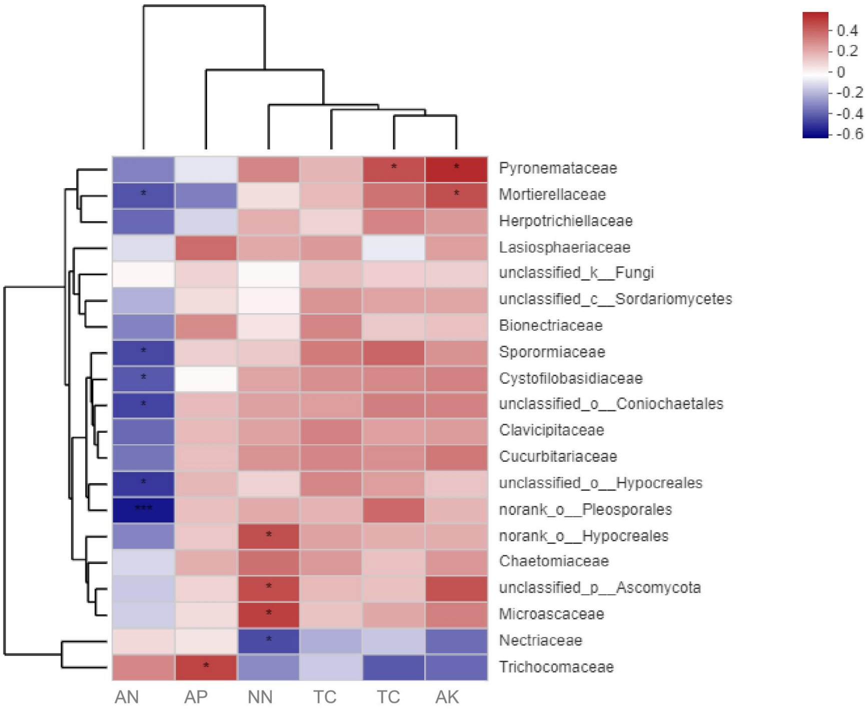

(b) Fungi

Table S1 ADONIS variation test of microbial alpha diversity treated with different level of ISMB based on the Bray-Curtis distance matrix.

Alpha Diversity	Dissimilarity	Bacteria P	Fungi P
Chao	Low ISMB vs Moderate ISMB	0.00	0.00
	Low ISMB vs High ISMB	0.00	0.00
	Moderate ISMB vs High ISMB	0.35	0.88
	None fertilizer vs Chemical fertilizer	1.00	0.96
	None fertilizer vs Biogas residues	0.89	0.96
	Chemical fertilizer vs Biogas residues	0.87	1.00
Shannon	Low ISMB vs Moderate ISMB	0.00	0.22
	Low ISMB vs High ISMB	0.00	0.04
	Moderate ISMB vs High ISMB	0.70	0.61
	None fertilizer vs Chemical fertilizer	0.95	0.84
	None fertilizer vs Biogas residues	0.68	0.96
	Chemical fertilizer vs Biogas residues	0.86	0.95

$p < 0.05$ indicates a significant difference.

Table S2 Paired Samples Test of SB and SR on alpha diversity index of bacteria and fungi

		Correlation	P
Bacteria chao	SB & SR	0.93	0.00
Bacteria shannon	SB & SR	0.85	0.00
Fungi chao	SB & SR	0.62	0.07
Fungi shannon	SB & SR	-0.02	0.97
		T	P (2-tailed)
Bacteria chao	SB - SR	-0.07	0.94
Bacteria shannon	SB - SR	-0.76	0.47
Fungi chao	SB - SR	2.10	0.07
Fungi shannon	SB - SR	1.88	0.10

$p < 0.05$ indicates a significant difference.

Table S3 Treatment effects on microbial alpha diversity

Sample	Bacteria				Fungi			
	Chao	Shannon	Increase index$_{based\ native\ soil}$ (%)	Increase index of O/C (%)	Chao	Shannon	Increase index$_{based\ native\ soil}$ (%)	Increase index of O/C (%)
Native soil	2792.47	6.73	/	/	383.44	3.06	/	/
NL_{SB}	778.6	4.31	-72.12%	/	166.17	2.18	-56.66%	/
CL_{SB}	1449.15	4.72	-48.11%	/	308.83	3.01	-19.46%	/
OL_{SB}	1492	5.33	-46.57%	2.96%	170.19	2.24	-55.61%	-44.89%
NM_{SB}	2731.24	5.8	-2.19%	/	437.25	3.07	14.03%	/
CM_{SB}	2561.94	6.34	-8.26%	/	396.92	2.41	3.52%	/
OM_{SB}	2923.74	6.66	4.70%	14.12%	390.37	2.82	1.81%	-1.65%
NH_{SB}	2755.44	5.95	-1.33%	/	316.63	2.38	-17.42%	/
CH_{SB}	2695.27	6.2	-3.48%	/	413.9	2.71	7.94%	/
OH_{SB}	2868.53	6.66	2.72%	6.43%	361.82	2.95	-5.64%	-12.58%
NL_{SR}	1149.96	4.62	-58.82%	/	121.07	1.26	-68.43%	/
CL_{SR}	766.23	4.47	-72.56%	/	35.2	0.84	-90.82%	/
OL_{SR}	1517.17	5.24	-45.67%	98.00%	126.07	1.69	-67.12%	258.15%
NM_{SR}	2786.39	6.55	-0.22%	/	281	2	-26.72%	/
CM_{SR}	2733.3	6.44	-2.12%	/	306.66	2.84	-20.02%	/
OM_{SR}	2593.24	5.87	-7.13%	-5.12%	291.11	1.57	-24.08%	-5.07%
NH_{SR}	2905.51	6.62	4.05%	/	348.09	2.61	-9.22%	/
CH_{SR}	2783.91	6.62	-0.31%	/	323	3.08	-15.76%	/
OH_{SR}	3091.09	6.64	10.69%	11.03%	459.65	2.91	19.88%	42.31%

Note：O/C means biogas residues treatment/chemical fertilizer treatment in different level ISMB and soil habitat.

Table S4 KO information of treatment for the Supplementary Figure S3

KO	Description
K00059	3-oxoacyl-[acyl-carrier protein] reductase [EC:1.1.1.100]
K00626	ABC-2 type transport system ATP-binding protein
K00799	ABC-2 type transport system permease protein
K01990	acetyl-CoA C-acetyltransferase [EC:2.3.1.9]
K01992	ATP-binding cassette, subfamily B, bacterial
K01999	branched-chain amino acid transport system substrate-binding protein
K02003	cold shock protein (beta-ribbon, CspA family)
K02004	glutathione S-transferase [EC:2.5.1.18]
K02014	iron complex outermembrane recepter protein
K02032	LacI family transcriptional regulator
K02033	methyl-accepting chemotaxis protein
K02034	peptide/nickel transport system ATP-binding protein
K02035	peptide/nickel transport system permease protein
K02529	peptide/nickel transport system permease protein
K03088	peptide/nickel transport system substrate-binding protein
K03406	putative ABC transport system ATP-binding protein
K03704	putative ABC transport system permease protein
K06147	RNA polymerase sigma-70 factor, ECF subfamily
K07090	serine/threonine protein kinase, bacterial [EC:2.7.11.1]
K08884	uncharacterized protein

Table S5 Topological indices of network under low, moderate and high ISMB level for bacteria in Fig. 5

	Phylum	Genus	Closness centrality	Betweeness centrality	Hub
L	*Bacteroidetes*	*Adhaeribacter*	0.61	2.00	0.23
		norank_f__Cytophagaceae	1.00	79.82	0.42
	Firmicutes	*Fictibacillus*	0.61	0.82	0.25
		Paenisporosarcina	0.65	5.40	0.27
		Tumebacillus	1.00	79.82	0.42
	Proteobacteria	*Massilia*	0.60	0.17	0.23
		Microvirga	0.61	0.82	0.25
		unclassified_f__Oxalobacteraceae	0.65	4.75	0.28
M	*Acidobacteria*	*unclassified_f__FamilyI_o__SubsectionIII*	0.63	20.48	0.22
	Actinobacteria	*norank_c__KD4-96*	0.65	22.46	0.22
		norank_f__Gemmatimonadaceae	0.63	11.45	0.21
	Proteobacteria	*norank_f__Rhodospirillaceae*	0.63	11.45	0.21
		norank_f__Longimicrobiaceae	0.65	28.07	0.21
		norank_f__OM1_clade	0.64	25.88	0.22
		norank_o__Acidimicrobiales	0.67	42.84	0.23
		unclassified_f__Intrasporangiaceae	0.65	28.07	0.21
		unclassified_o__Sphingomonadales	0.65	25.04	0.21
H	*Actinobacteria*	*Blastococcus*	0.49	42.29	0.21
		Gaiella	0.49	21.59	0.26
		norank_f__OM1_clade	0.46	32.19	0.27
		norank_o__Gaiellales	0.50	39.38	0.28
		Pseudarthrobacter	0.42	19.76	0.23
	Chloroflexi	*norank_f__Anaerolineaceae*	0.49	81.62	0.22
		Roseiflexus	0.48	17.94	0.24
	Gemmatimonadetes	*norank_f__Gemmatimonadaceae*	0.53	173.05	0.26
	Nitrospirae	*Nitrospira*	0.45	29.12	0.25
	Proteobacteria	*norank_f__Nitrosomonadaceae*	0.49	43.37	0.26
		norank_f__Rhodobiaceae	0.48	12.90	0.25
		Microvirga	0.48	10.12	0.23
		norank_f__BIrii41	0.45	42.54	0.26

Table S6 Topological indices of network under low, moderate and high ISMB level for fungi in Fig. 5

Treatment	Phylum	Family	Closness centrality	Betweeness centrality	Hub
L	*Ascomycota*	*Chaetomiaceae*	0.53	0.00	0.25
		Davidiellaceae	0.53	0.00	0.25
		Nectriaceae	0.90	29.33	0.61
		unclassified_c__Sordariomycetes	0.60	3.50	0.34
		unclassified_p__Ascomycota	0.60	3.50	0.34
	Basidiomycota	*Cystofilobasidiaceae*	0.60	0.33	0.38
M	*Ascomycota*	*Cordycipitaceae*	0.61	10.79	0.21
		Nectriaceae	0.62	26.68	0.23
		norank_p__Ascomycota	0.67	33.97	0.28
		Pyronemataceae	0.63	33.45	0.22
		Trichocomaceae	0.62	31.31	0.21
	Basidiomycota	*Cystofilobasidiaceae*	0.67	30.66	0.27
H	*Ascomycota*	*Chaetothyriaceae*	0.91	21.74	0.20
		Coniochaetaceae	0.95	23.74	0.21
		Davidiellaceae	0.95	23.94	0.21
		Gymnoascaceae	0.93	19.82	0.21
		norank_o__Eurotiales	0.93	23.72	0.21
		norank_p__Ascomycota	0.95	23.00	0.22
		Pseudeurotiaceae	0.95	24.60	0.21
	Chytridiomycota	*Rhizophlyctidaceae*	1.00	26.78	0.22

Table S7 Topological indices of network under none, chemical and biogas residues three fertilizer type for bacteria in Fig. 5

	Phylum	Genus	Closness centrality	Betweeness centrality	Hub
N	*Bacteroidetes*	*Adhaeribacter*	0.41	2.24	0.31
		Flavisolibacter	0.51	138.05	0.31
		norank_f__Chitinophagaceae	0.41	2.24	0.31
	Firmicutes	*Bacillus*	0.42	5.56	0.32
		Paenibacillus	0.40	1.71	0.28
	Proteobacteria	*Bdellovibrio*	0.41	2.24	0.31
		Devosia	0.43	18.82	0.31
		Ensifer	0.41	18.08	0.28
		Massilia	0.43	18.82	0.31
		Paucimonas	0.41	2.39	0.30
		Rhizobium	0.33	0.00	0.23
O	*Actinobacteria*	*norank_o__Acidimicrobiales*	0.52	142.58	0.30
	Bacteroidetes	*norank_f__Cytophagaceae*	0.48	17.69	0.26
	Chloroflexi	*norank_o__AKYG1722*	0.48	86.29	0.28
	Firmicutes	*Bacillus*	0.48	28.26	0.27
		Paenibacillus	0.52	231.30	0.24
		Paenisporosarcina	0.47	28.82	0.22
	Gemmatimonadetes	*norank_c__Gemmatimonadetes*	0.43	12.29	0.21
	Proteobacteria	*Devosia*	0.47	13.35	0.26
		Ensifer	0.47	13.77	0.26
		Massilia	0.51	79.14	0.30
		Rhizobium	0.46	17.79	0.25
C	*Bacteroidetes*	*Adhaeribacter*	0.50	49.14	0.31
		Pedobacter	0.47	9.30	0.21
	Firmicutes	*Bacillus*	0.47	9.30	0.21
		Paenibacillus	0.46	11.42	0.27
		Paenisporosarcina	0.55	55.52	0.29
	Proteobacteria	*Brevundimonas*	0.41	0.00	0.20
		Devosia	0.42	2.67	0.25
		Ensifer	0.50	28.89	0.33
		Massilia	0.51	81.12	0.20
		Rhizobium	0.42	2.67	0.25
	Verrucomicrobia	*Chthoniobacter*	0.49	12.94	0.30

Table S8 Topological indices of network under none, chemical and biogas residues three fertilizer type for fungi in Fig. 5

	Phylum	family	Closness centrality	Betweenes scentrality	Hub
N	*Ascomycota*	*Clavicipitaceae*	0.42	33.54	0.30
		Cucurbitariaceae	0.45	17.92	0.31
		Microascaceae	0.33	0.00	0.21
		Myxotrichaceae	0.47	38.12	0.32
		norank_c__Sordariomycetes	0.44	10.08	0.28
		norank_o__Hypocreales	0.43	8.41	0.40
		norank_o__Pleosporales	0.37	0.20	0.22
		Sporormiaceae	0.40	1.95	0.30
		unclassified_o__Hypocreales	0.43	8.41	0.40
	Zygomycota	*Mortierellaceae*	0.41	3.38	0.32
O	*Ascomycota*	*Cucurbitariaceae*	0.77	2.63	0.38
		Lasiosphaeriaceae	0.71	0.80	0.36
		Myxotrichaceae	0.56	0.00	0.27
		norank_o__Pleosporales	0.71	0.80	0.36
		Trichocomaceae	0.83	17.33	0.35
		unclassified_o__Hypocreales	0.77	2.63	0.38
		unclassified_p__Ascomycota	0.67	0.00	0.32
	Basidiomycota	*Cystofilobasidiaceae*	0.71	0.80	0.36
C	*Ascomycota*	*Chaetomiaceae*	0.78	8.00	0.44
		Cucurbitariaceae	0.64	6.00	0.25
		Nectriaceae	0.78	4.67	0.48
		unclassified_p__Ascomycota	0.54	0.00	0.35
	Basidiomycota	*Cystofilobasidiaceae*	0.64	0.67	0.43
	Zygomycota	*Mortierellaceae*	0.64	0.67	0.43

Table S9 Topological indices of network of bulk soil and rhizosphere soil for bacteria in Fig. 5

	Phylum	Genus	Closness centrality	Betweeness centrality	Hub
SR	*Bacteroidetes*	*Adhaeribacter*	0.61	3.33	0.25
		Flavisolibacter	0.59	1.98	0.23
		Pedobacter	0.60	2.02	0.23
	Cyanobacteria	*norank_c__Cyanobacteria*	0.68	75.35	0.26
	Firmicutes	*Bacillus*	0.63	11.13	0.24
		Paenibacillus	0.60	8.43	0.21
		Paenisporosarcina	0.66	6.99	0.26
	Proteobacteria	*Bdellovibrio*	0.63	6.66	0.25
		Brevundimonas	0.61	2.49	0.24
		Devosia	0.63	5.20	0.25
		Ensifer	0.66	26.90	0.24
		Massilia	0.64	31.24	0.25
		Pseudomonas	0.61	4.53	0.24
		Rhizobium	0.64	6.49	0.26
		unclassified_f__Oxalobacteraceae	0.66	6.99	0.26
SB	*Actinobacteria*	*Pseudarthrobacter*	0.64	43.35	0.20
	Bacteroidetes	*Adhaeribacter*	0.68	15.22	0.25
		Flavisolibacter	0.70	6.34	0.26
	Firmicutes	*Bacillus*	0.74	11.34	0.28
		Paenibacillus	0.70	8.06	0.27
		Paenisporosarcina	0.72	8.37	0.27
	Proteobacteria	*Devosia*	0.74	10.88	0.28
		Ensifer	0.72	9.09	0.27
		Lysobacter	0.61	1.47	0.22
		Massilia	0.72	20.48	0.26
		Paucimonas	0.68	4.12	0.25
		Rhizobium	0.66	7.81	0.27
		Sphingomonas	0.66	3.90	0.25

Table S10 Topological indices of network of bulk soil and rhizosphere soil for fungi in Fig. 5

	Phylum	Family	Closness centrality	Betweeness centrality	Hub
SR	*Ascomycota*	*Chaetomiaceae*	0.80	0.50	0.35
		Cucurbitariaceae	0.80	0.50	0.35
		Lasiosphaeriaceae	1.00	3.33	0.41
		Nectriaceae	0.67	0.00	0.25
		norank_o__Pleosporales	0.67	0.00	0.25
		unclassified_c__Sordariomycetes	0.80	0.50	0.35
		unclassified_p__Ascomycota	0.89	1.83	0.38
	Basidiomycota	*Cystofilobasidiaceae*	1.00	3.33	0.41
SB	*Ascomycota*	*Clavicipitaceae*	0.51	28.46	0.31
		Cucurbitariaceae	0.48	23.43	0.24
		Nectriaceae	0.55	67.74	0.20
		norank_o__Eurotiales	0.49	12.23	0.29
		norank_o__Pleosporales	0.46	7.45	0.25
		norank_p__Ascomycota	0.54	34.63	0.30
		Sporormiaceae	0.43	22.15	0.23
		unclassified_o__Hypocreales	0.45	4.72	0.23
	Basidiomycota	*Cystofilobasidiaceae*	0.55	73.11	0.31
	unclassified_k__Fungi	*unclassified_k__Fungi*	0.52	23.12	0.26
	Zygomycota	*Mortierellaceae*	0.54	55.54	0.33
